# VAR2CSA-specific IgG and IgM antibodies are markers of exposure and protection against adverse malaria pregnancy outcomes

**DOI:** 10.1186/s12936-025-05773-0

**Published:** 2025-12-31

**Authors:** Akachukwu M. Onwuka, Elizabeth H. Aitken, Wina Hasang, Mwayiwawo Madanitsa, Victor Mwapasa, Kamija Phiri, Feiko O. ter Kuile, Stephen J. Rogerson

**Affiliations:** 1 Department of Infectious Diseases, The Peter Doherty Institute of Infection and Immunity, University of Melbourne, Melbourne, VIC 3000 Australia; 2 Department of Microbiology and Immunology, The Peter Doherty Institute of Infection and Immunity, University of Melbourne, Melbourne, VIC 3000 Australia; 3 Department of Clinical Sciences, Academy of Medical Sciences, Malawi University of Science and Technology, Thyolo, Malawi; 4 Department of Clinical Sciences, Liverpool School of Tropical Medicine, Liverpool, UK; 5 Department of Epidemiology and Biostatistics, School of Global and Public Health, Kamuzu University of Health Sciences, Blantyre, Malawi; 6 Training and Research Unit of Excellence, Blantyre, Malawi; 7 Department of Medicine (RMH), The Peter Doherty Institute of Infection and Immunity, University of Melbourne, Melbourne, VIC 3000 Australia

**Keywords:** Placental malaria, VAR2CSA, Low birth weight, Small for gestational age, Preterm delivery, Maternal anaemia

## Abstract

**Background:**

Placental malaria is caused by the binding of the *Plasmodium falciparum* erythrocyte membrane protein 1 (*Pf*EMP1) protein VAR2CSA, found on the surface of infected erythrocytes, to placental tissue. Complications include maternal anaemia, low birth weight, small for gestational age and preterm delivery. Acquisition of antibodies against VAR2CSA during pregnancy has been linked to immunity against infection.

**Methods:**

Pregnant Malawian women were enrolled at their first antenatal care visit at 16–28 weeks’ gestation into a trial of malaria prevention. Women with malaria infection at enrolment (n = 321) or any later point in pregnancy (n = 145) were selected. The IgG and IgM plasma levels against the VAR2CSA DBL1X-ID2a domain were measured at enrolment and delivery. Associations between the DBL1X-ID2a VAR2CSA-specific IgG and IgM antibody levels at enrolment or delivery and low birth weight, small for gestational age, maternal anaemia at delivery, and preterm delivery were assessed using logistic regression with confounder adjustment.

**Result:**

Women with malaria infection at enrolment had higher antibody levels to DBL1X-ID2a than uninfected women, and these declined from enrolment to delivery. There were no significant associations between the IgG antibody level measured at enrolment and the birth outcomes of interest, but the IgG antibody level at delivery in women uninfected at enrolment was associated with a lower risk of low birth weight, adjusted odds ratio (aOR) 0.43 (95% CI 0.19–0.97) *p* = 0.04. Additionally, in women infected at enrolment, one log higher IgM antibodies to DBL1X-ID2a VAR2CSA at enrolment were associated with a significant 23% decrease in maternal anaemia at delivery, aOR 0.77 (95% CI 0.60–0.99), *p* = 0.04.

**Conclusion:**

VAR2CSA-specific IgG and IgM antibodies are markers of malaria infection and protection against placental malaria outcomes.

**Supplementary Information:**

The online version contains supplementary material available at 10.1186/s12936-025-05773-0.

## Introduction

The 2023 World Malaria Report recorded 263 million cases and 597,000 malaria deaths across 85 countries [1]. Pregnant women and children under five years old are the most vulnerable groups to malaria infection [2, 3].

Malaria in pregnancy (MiP) leads to infection of the placenta, known as placental malaria (PM), which increases the risk of placental damage and dysfunction [4]. PM is caused by the sequestration of infected erythrocytes (IEs) in the intervillous space of the placenta [5, 6]. VAR2CSA, a *Plasmodium falciparum* erythrocyte membrane protein 1 (PfEMP1) variant that binds to chondroitin sulfate A (CSA) on the syncytiotrophoblast, mediates the sequestration of IEs in the placenta [7]. VAR2CSA is a 350 kDa transmembrane protein composed of six Duffy-Binding-Like (DBL) domains and several inter-domain regions; of these the DBL2 domain and flanking sequences have been implicated in CSA binding [8] and developed as potential vaccine candidates [9, 10].

VAR2CSA plays a crucial role in the pathogenesis of PM, but evidence suggesting its specific targeting by protective antibodies has been inconsistent. Antibodies against VAR2CSA are acquired during pregnancy with exposure to *P. falciparum* [11]. Primigravid women and men in malaria-endemic areas typically have few antibodies specific to VAR2CSA, but these levels rise in women as the number of pregnancies increases [12, 13]. Antibodies to VAR2CSA recombinant proteins or VAR2CSA expressed on IEs have been reported to be associated with a reduced risk of placental infection or higher birthweights [14, 15]. However, a systematic review by Cutts, Agius et al. [16] found that antibody responses to VAR2CSA are often markers of malaria infection in pregnancy rather than protection from infection.

Although IgG responses to VAR2CSA have been widely studied, IgM, the first antibody isotype produced during malaria infection, which may contribute to early parasite clearance through complement activation and agglutination [17], remains poorly studied. Measuring VAR2CSA-specific IgM alongside IgG therefore provides a more comprehensive understanding of humoral immunity in pregnancy-associated malaria. It was hypothesized that pregnant women living in malaria-endemic Malawi and infected with *P. falciparum* naturally acquired immunoglobulin G and M (IgG and IgM) antibodies against the DBL1X-ID2a VAR2CSA antigen [18] and that these could be associated with protection from PM and adverse birth outcomes. For this analysis, infection at enrolment was evaluated as the primary exposure, as this was the only time point for which its association with antibody acquisition was assessed. To explore this, plasma samples from the IMPROVE cohort, a clinical trial evaluating malaria preventive treatment during pregnancy in a malaria-endemic region of Malawi, were used. IgG and IgM antibodies were measured at enrolment and delivery and their associations with adverse pregnancy outcomes were analyzed.

## Materials and methods

### Ethics approval and consent to participate

Ethics approval was obtained from the ethics committees of the Kenya Medical Research Institute, Nairobi, Kenya; the College of Medicine, Blantyre, Malawi; the Medical Research Coordinating Committee, Dar Es Salaam, Tanzania; the Liverpool School of Tropical Medicine (LSTM), Liverpool, UK and Melbourne Health Human Research Ethics Committee. All study participants gave written informed consent.

### Study population and sample collection

A detailed description of the IMPROVE trial, a randomised, double-blinded, three-arm, partially placebo-controlled trial of intermittent preventive treatment in pregnancy (IPTp), has been reported [19]. In Kenya, Tanzania, and Malawi, HIV-negative women with viable singleton pregnancies between 16 and 28 weeks of gestation and who provided informed consent were enrolled in the study and followed up until delivery. Participants received IPTp with sulfadoxine-pyrimethamine (SP), dihydroartemisinin-piperaquine (DP) or DP in combination with an initial dose of azithromycin (DP + AZ) at least four weeks apart in the second and third trimesters of pregnancy. The current study made use of samples from a subgroup of participants who were known to be infected at study entry or infected later in pregnancy from antenatal clinics in Chikwawa, Madziabango, Mangochi, Mpemba, and Zomba in Malawi.

A venous or finger-prick whole blood sample was collected for malaria diagnosis via microscopy and quantitative polymerase chain reaction (qPCR) at each study visit. Participants who tested positive for *P. falciparum* infection at enrolment by PCR and/or light microscopy were classified as ‘infected at enrolment’, those who were negative by both PCR and light microscopy at enrolment but who became infected later in the pregnancy were classified as ‘uninfected at enrolment’. Demographic and clinical data collected at enrolment included maternal gravidity, age (years), height (cm), mid-upper arm circumference (MUAC, cm; assessed at the midpoint between the tips of the shoulder and elbow using a flexible tape measure [20], residence, and bed net use (yes or no). Maternal haemoglobin levels were recorded at enrolment and delivery and anaemia was defined as a haemoglobin level < 11.0 g/dL. At birth, the newborn’s weight was measured immediately using a digital scale and the gestational age (days) and sex of the baby (male or female) were documented. If the newborn’s weight was measured postpartum, it was adjusted to estimate the approximate weight at delivery [21].

Pregnant women were classified at enrolment as first-time mothers (primigravidae), those with one previous pregnancy (secundigravidae), or those with two or more previous pregnancies (multigravidae). Plasma samples taken at enrolment (16–28 weeks) and delivery were frozen, shipped on dry ice to the laboratory, and stored at − 80 °C upon arrival for further use. Paired enrolment and delivery samples were tested together to determine whether antibody levels changed during pregnancy.

### Recombinant protein

Recombinant VAR2CSA protein DBL1X-ID2a, which has been reported as the minimal CSA-binding region [18], was used for this study. It is a recombinant protein expressed in *Escherichia coli* [22], and was a gift from A/Prof Morten Nielsen at the University of Copenhagen.

### Measurement of plasma antibody levels against recombinant proteins

Plasma levels of IgG and IgM antibody against DBL1X-ID2a VAR2CSA were measured using an Enzyme-Linked Immunosorbent Assay (ELISA). In brief, the wells of microtiter plates (F96 MaxiSorp Nunc immunoplates, Thermo Fisher Scientific, Waltham, MA, USA) were coated with 50 µL/well of 2 µg/mL recombinant DBL1X-ID2a VAR2CSA protein prepared in phosphate-buffered saline (PBS) and incubated overnight at 4 °C. The next day, the plate was washed twice with PBS to remove unbound protein, and the plate was further blocked for 1 h at room temperature (RT) with 180 μL/well blocking buffer (PBS, 0.05% Tween 20, and 1% bovine serum albumin, or BSA). Excess blocking buffer was removed by washing thrice with wash buffer (PBS, 0.05% Tween 20). For positive controls, human pooled positive sera IgG standards (PPS-IgG) and human pooled positive sera IgM standards (PPS-IgM) pooled from individuals identified as having high IgG and IgM reactivity to DBL1X-ID2a VAR2CSA respectively were sourced from malaria-endemic regions and prepared as serial dilutions ranging from 1:1000 to 1:64000. For negative controls, plasma samples from malaria-naïve residents of Melbourne, Australia were used. Heat-inactivated plasma samples were diluted 1:4000 and 1:1000 in blocking buffer for IgG and IgM studies, respectively, and 50 µL were plated in duplicate wells, and incubated for 2 h at RT. The plates were then washed five times with the wash buffer. Bound IgG and IgM were detected using biotinylated goat anti-human IgG (Mabtech, Sweden; Catalogue number (Cat no) 3830-4-250) or IgM (Mabtech, Sweden; Cat no 3880-6-250) prepared in blocking buffer at concentrations of 0.5 µg/mL per well (1:1000 dilution) and incubated for 1 h at RT before washing five times. Bound biotin was detected by incubating with 50 µL/well of streptavidin–horseradish peroxidase (R&D Systems, Cat no DY998) 1:200 for 1 h at RT. The plate was washed five times, as previously described. To measure bound horseradish peroxidase, substrate solution TMB (3, 3’,5,5’-tetramethylbenzidine, 50 µL/well, BD Biosciences, USA; Cat no 555214) was prepared according to the manufacturer’s instructions and colour development was stopped with 50 μL/well 2 N H_2_SO_4_ after 5 to 10 min at RT. The optical density (OD) was measured at 450 nm using an automated ELISA microplate reader (FLUOstar Omega, BMG LABTECH), and OD values were generated for each sample. Before plotting the standard curve, the OD was calculated by subtracting the background value of the uncoated well from the values of the VAR2CSA-coated wells and the mean value was recorded. All samples were tested in duplicate and samples whose duplicates showed more than 20% mean variance were re-run. Antibody levels were expressed as arbitrary units (AU) relative to the standard curve.

### Variable definitions

Participants were grouped by infection status at enrolment. Potential key confounders considered include gravidity, maternal age, maternal height, and MUAC. Adverse birth outcomes or complications of malaria in pregnancy included maternal anaemia (haemoglobin concentration at delivery < 11 g/dL), low birth weight (LBW; weight of baby < 2500 g), preterm delivery (PTD; before 37 weeks of gestation), and small for gestational age (SGA; weight below the 10th percentile for the infant’s gestational age) [4, 23, 24].

Z-scores for newborn birth size were computed using the INTERGROWTH reference population for newborn size[25]. SGA was defined as a growth z-score < − 1.28 [19].

### Statistical analysis

All data were entered into Microsoft Excel, and statistical analysis was performed using Stata Version 17.0 (StataCorp, College Station, Texas 77845 USA) and GraphPad Prism 10. Before analysis, AU of IgG and IgM against VAR2CSA were logarithmically transformed (natural log, ln) to normalize the data. Values ≤ 1 were adjusted to 1 before logarithmic transformation. Data were presented as % (number/total) for categorical variables and means ± standard deviation (SD) for continuous variables. Maternal age, gravidity, MUAC and maternal height were assessed as potential confounders based on previous findings in the study area [19]. To investigate the relationship between immunity and outcomes of placental malaria, the natural logarithms (ln) of IgG or IgM measurement against DBL1X-ID2a VAR2CSA at enrolment or delivery were considered the exposure variables. Univariate logistic regression was utilised to ascertain the relationship between (ln) IgG and IgM antibody levels against DBL1X-ID2a VAR2CSA and various pregnancy outcomes such as LBW, PTD, SGA infants, and maternal anaemia. Multivariate logistic regression was conducted to investigate the association between the exposure (ln antibody levels) at enrolment and delivery and pregnancy outcomes while adjusting for confounding factors. *P*-values of less than 0.05 were considered statistically significant. In addition, linear regression analyses were conducted to determine factors associated with levels of IgG or IgM antibodies to DBL1X-ID2a VAR2CSA at enrolment and at delivery. The IgG/IgM ratio was calculated for each sample by dividing the measured IgG concentration by the corresponding IgM concentration and subsequently natural log–transformed (ln IgG/IgM). Multivariate logistic regression analyses were then conducted to examine the association between ln IgG/IgM and pregnancy outcomes.

## Results

The IMPROVE trial participants who contributed plasma samples to this study comprised 466 pregnant Malawian women who were infected at some point during their pregnancy. Of these, 321 women had *P. falciparum* infection at enrolment while 145 were infected later in pregnancy (Table 1). The mean ± SD age was 22.6 ± 5.1 and 24.6 ± 6.1 years for infected and uninfected women at enrolment, respectively. Approximately 38.0% (177/466) of women were primigravid, with the majority residing in Chikwawa and Mangochi.

**Table 1:** Demographic and clinical characteristics of the study population stratified by malaria infection status at enrolment

Characteristics	Total n = 466	Infected at enrolment n = 321 (%)	Uninfected at enrolment n = 145 (%)
Age, years	23.2 ± 5.5	22.6 ± 5.1	24.6 ± 6.1
Gestational age at enrolment, days	148.7 ± 21.4	149.0 ± 21.7	148.1 ± 21.0
Gravidity^a^			
Primigravid	177 (38.1)	127 (39.7)	50 (34.7)
Secundigravid	120 (25.9)	87 (27.2)	33 (22.9)
Multigravid	167 (36.0)	106 (33.1)	61 (42.4)
Location of study			
Chikwawa	133 (28.7)	88 (27.4)	45 (31.0)
Madziabango	72 (15.5)	56 (17.5)	16 (11.0)
Mangochi	133 (28.7)	109 (34.0)	24 (16.6)
Mpemba	63 (13.6)	44 (13.7)	19 (13.1)
Zomba	65 (14.0)	24 (7.5)	41 (28.3)
Intervention			
SP	155 (33.3)	97 (30.2)	58 (40.0)
DP	150 (32.2)	103 (32.1)	47 (32.4)
DP + AZ	161 (34.5)	121 (37.7)	40 (27.6)
Net use^b^			
Yes	258 (55.4)	162 (50.6)	96 (66.2)
No	207 (44.4)	158 (49.4)	49 (33.8)
Smokes^b^			
Yes	2 (0.4)	2 (0.6)	0
No	463 (99.6)	318 (99.4)	145 (100.0)
Maternal weight at enrolment (kg)	57.9 ± 8.2	57.2 ± 8.1	59.3 ± 8.3
Maternal height, (cm)^b^	157.3 ± 6.6	157.0 ± 6.8	158.2 ± 5.8
MUAC at enrolment (cm)^b^	26.1 ± 3.0	25.9 ± 3.0	26.5 ± 3.0
Anaemia at enrolment^c^			
Yes	239 (52.2)	186 (57.9)	53 (36.6)
No	219 (47.8)	131 (40.8)	88 (60.7)
Hb at enrolment^c^ (g/dL)	10.9 ± 1.5	10.6 ± 1.4	11.3 ± 1.4
Birth outcomes			
Anaemia at delivery^d^			
Yes	102 (25.3)	72 (26.9)	30 (22.2)
No	301 (74.7)	196 (73.1)	105 (77.8)
Hb at delivery, g/dL^d^	11.8 ± 1.5	11.7 ± 1.4	11.9 ± 1.5
Gestational age at delivery, (days)^e^	273.9 ± 16.6	272.8 ± 16.1	275.2 ± 19.0
LBW^f^			
Yes	45 (10.9)	32 (11.7)	13 (9.4)
No	366 (89.1)	241 (88.3)	125 (90.6)
SGA^g^			
Yes	92 (22.8)	59 (21.9)	33 (24.6)
No	312 (77.2)	211 (78.1)	101 (75.4)
Foetal loss^h^			
Yes	9 (2.1)	5 (1.8)	4 (2.8)
No	416 (97.9)	278 (98.2)	138 (97.2)
Preterm delivery^e^			
Yes	38 (9.0)	27 (9.6)	11 (7.9)
No	383 (91.0)	254 (90.4)	129 (92.1)
Sex of baby^i^			
Male	193 (46.6)	125 (45.5)	68 (48.9)
Female	221 (53.4)	150 (54.5)	71 (51.1)

Data represented as mean ± standard deviation or number (percentage)

*SP* sulfadoxine-pyrimethamine, *DP* dihydroartemisinin-piperaquine, *AZ* azithromycin, *MUAC* middle upper arm circumference, *Hb* haemoglobin concentration, Anaemia, Hb concentration below 11.0 g/dL, *LBW* weight < 2.5 kg, *SGA* small for gestational age (weight z-score < − 1.28)

^a^Two participants with missing data on gravidity

^b^One participant with missing data on the net use, smoking status, and maternal height

^c^eight participants with missing data at enrolment on Hb level and anaemic status

^d^sixty-three participants with missing data at delivery on Hb level and anaemic status

^e^forty-five participants with missing data on gestational age

^f^fifty-five missing data on low birth weight

^g^sixty-two missing data on small for gestational age

^h^forty-one missing data on fetal loss

^i^fifty-two missing sex of the baby

### Changes in IgG and IgM antibody levels during pregnancy

**Fig. 1:**
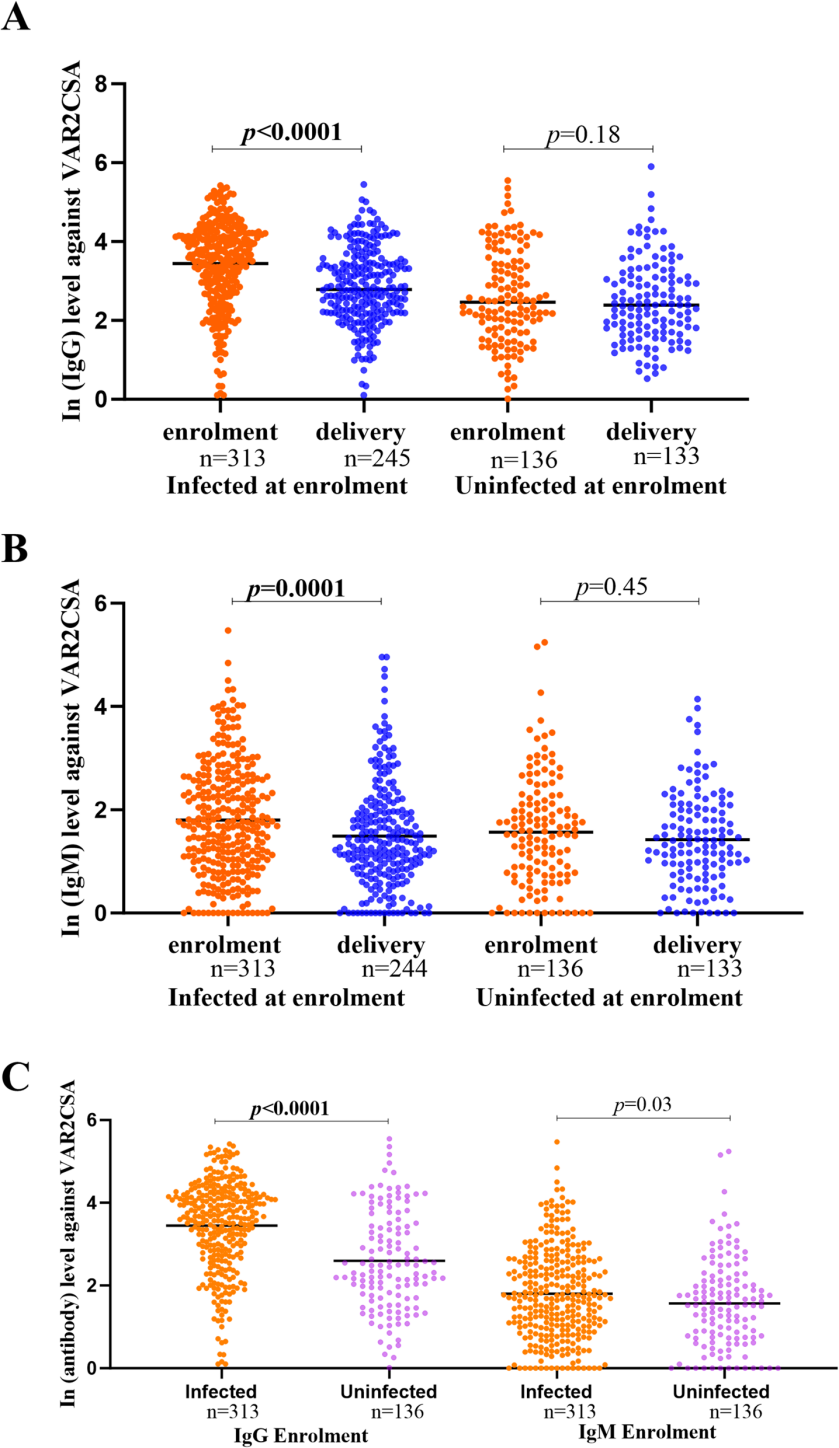
**A** ln (IgG), **B** ln (IgM) and **C** ln (antibody) levels at study enrolment and delivery. The Y axis represents ln (IgG) (**A**) or ln (IgM) (**B**) or ln (antibody) (**C**) levels against DBL1X-ID2a VAR2CSA. The X-axis shows the number of Malawian pregnant women tested at enrolment or delivery based on the presence or absence of malaria infection at enrolment. The black line represents the mean ln (IgG or IgM) antibody level. *P* value was determined using the non-parametric Wilcoxon signed-rank test (**A** and **B**) and Mann–Whitney U test (**C**)

For women infected at enrolment, IgG antibody levels (expressed as ln) decreased from enrolment (mean ± SD; 3.45 ± 1.09) to delivery (2.89 ± 0.97) (*p* < 0.0001) (Fig. 1A). Likewise, IgM antibody levels also demonstrated a significant reduction from enrolment (1.80 ± 0.06) to delivery (1.49 ± 0.07) (*p* = 0.0001) (Fig. 1B). For women uninfected at enrolment, IgG and IgM antibody levels did not differ significantly between enrolment and delivery (Fig. 1 A and B).

At enrolment, levels of IgG antibody to the recombinant VAR2CSA were higher in women who were infected with *P. falciparum* (3.45 ± 1.09) than uninfected women (2.60 ± 1.19) (*p* < 0.0001; Fig. 1C) and levels of IgM for infected women were also higher (1.80 ± 0.06) than in uninfected women at enrolment (1.57 ± 0.09) (*p* = 0.03) (Fig. 1C). IgG levels showed a modest gestational age–related increase at enrolment, with higher levels observed at 21–24 weeks compared with 16–20 weeks in both infected (*p* = 0.05) and uninfected women (*p* = 0.01). In contrast, IgM levels did not differ significantly across gestational age categories in either infected or uninfected women, indicating no gestational age–dependent pattern at enrolment (Supplementary Fig. 1).

### Effect of gravidity on VAR2CSA-specific antibody levels

**Fig. 2:**
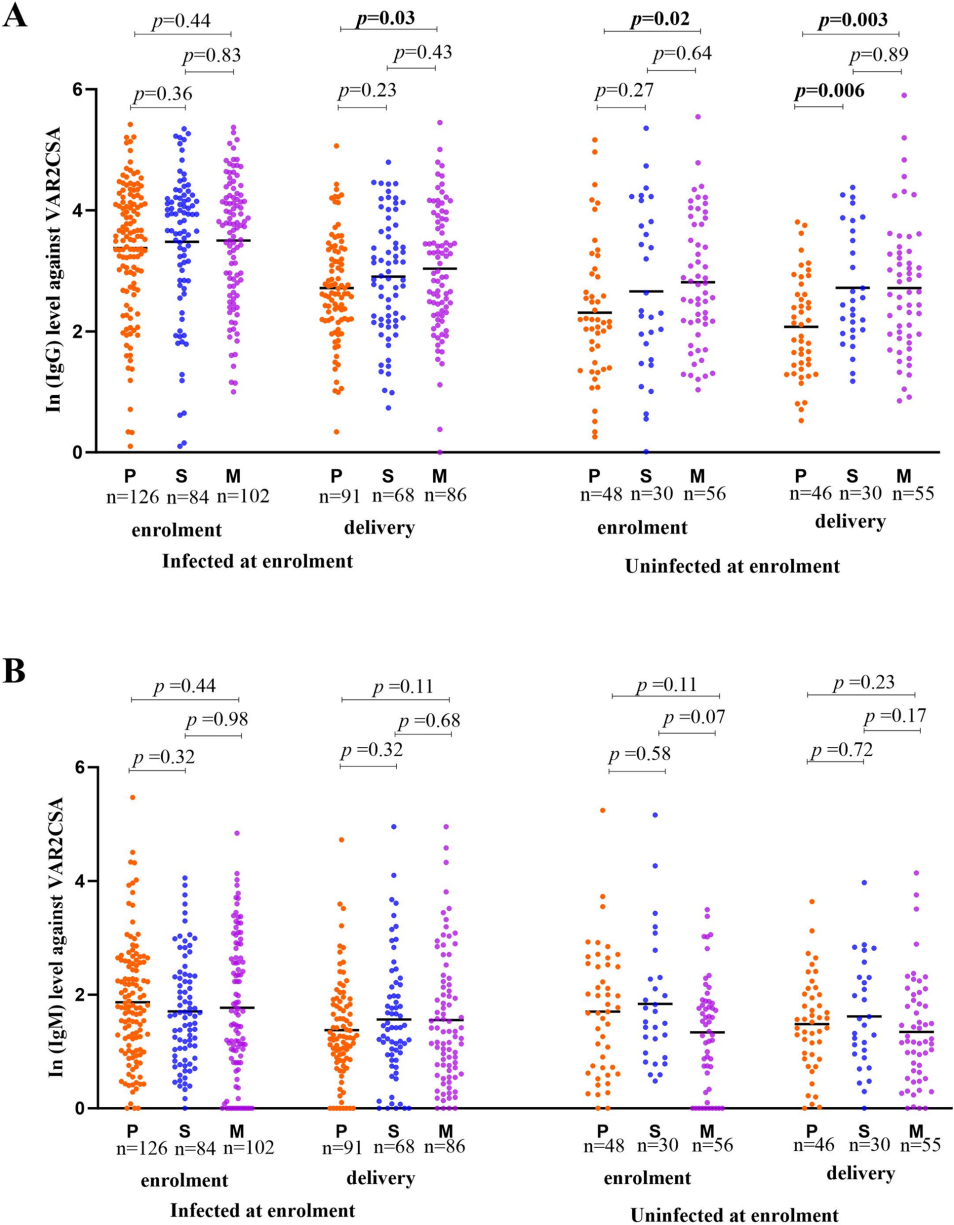
**A** ln (IgG) and **B** ln (IgM) antibody levels against DBL1X-ID2a VAR2CSA at study enrolment and delivery by gravidity. The Y-axis represents ln (IgG) or (IgM) levels against DBL1X-ID2a VAR2CSA at enrolment. The X-axis represents the classification of the women based on gravidity. P, S and M represent primigravid, secundigravid and multigravid women, respectively. *P* value was determined using the non-parametric Mann–Whitney U test. The black line represents the mean ln (IgG) or (IgM) antibody levels against DBL1X-ID2a VAR2CSA

Among women uninfected at enrolment, IgG antibody levels showed a significant increase with gravidity from (mean ± SD) 2.3 ± 1.13 in primigravid to 2.8 ± 1.06 in multigravid women (*p* = 0.02) (Fig. 2A). Among secundigravid women, uninfected women had significantly higher antibodies at enrolment (Coeff = 0.72, 95% CI 0.19–1.26, *p* = 0.008) and at delivery (Coeff = 0.58, 95% CI 0.13–1.03, *p* = 0.01), while infected women showed no significant difference. A similar pattern was observed in multigravid women, with uninfected participants maintaining higher antibody levels at enrolment (Coeff = 0.64, 95% CI 0.19–1.09, *p* = 0.005) and delivery (Coeff = 0.64, 95% CI 0.25–1.03, *p* = 0.001) (Table 2).

**Table 2:** Factors that may affect levels of in IgG antibody to DBL1X-ID2a VAR2CSA at enrolment and delivery

	ln IgG antibody at enrolment				ln IgG antibody at delivery				
	Infected at enrolment Coeff (95% CI)	*p-*value	Uninfected at enrolment Coeff (95% CI)	*p-*value	Infected at enrolment Coeff (95% CI)	*p-*value	Uninfected at enrolment Coeff (95% CI)	*p-*value	
^#^Secundigravid	0.10 (− 0.20, 0.41)	0.50	**0.72 (0.19, 1.26)**	**0.008**	0.02 (− 0.28, 0.33)	0.88	**0.58 (0.13, 1.03)**		**0.01**
^#^Multigravid	0.13 (− 0.16, 0.43)	0.38	**0.64(0.19, 1.09)**	**0.005**	0.05(− 0.25, 0.35)	0.75	**0.64 (0.25, 1.03)**		**0.001**
Maternal age(years)	− 0.12 (− 0.64, 0.40)	0.65	0.83 (− 0.06, 1.71)	0.07	0.43 (− 0.26, 1.11)	0.22	**1.08 (0.06, 2.09)**		**0.04**
Maternal height (cm)	− 0.06 (− 0.76, 0.63)	0.86	0.04 (− 0.79, 0.88)	0.92	− **0.98 (**− **1.87, **− **0.09)**	**0.03**	0.58 (− 0.40, 1.56)		0.24
MUAC (cm)	− 0.24 (− 0.54, 0.05)	0.11	0.04 (− 0.37, 0.45)	0.84	− 0.29 (− 0.62, 0.04)	0.08	0.25 (− 0.24, 0.75)		0.31
^+^Madziabango	0.82 (0.47, 1.18)	0.00	1.52 (0.86, 2.17)	0.00	0.52 (0.17, 0.89)	0.004	0.51 (− 0.13, 1.14)		0.12
^+^Mangochi	0.04 (− 0.35, 0.43)	0.83	0.77 (0.16, 1.39)	0.11	0.02 (− 0.40, 0.44)	0.92	0.44 (− 0.18, 1.06)		0.16
^+^Mpemba	0.04 (− 0.48, 0.56)	0.88	0.46 (− 0.04, 0.96)	0.07	0.004 (− 0.48, 0.46)	0.99	− 0.007 (− 0.45, 0.43)		0.98
^+^Zomba	0.42 (0.13, 0.72)	0.005	0.71 (0 0.15, 1.27)	0.01	− 0.02 (− 0.32, 0.28)	0.89	− 0.07 (− 0.59, 0.45)		0.78
*DP	0.009 (− 0.30, 0.32)	0.95	− 0.15 (− 0.63, 0.32	0.53	0.02 (− 0.28, 0.32)	0.88	− 0.23 (− 0.64, 0.19)		0.28
*DP + AZ	0.13 (− 0.16, 0.43)	0.38	0.29 (− 0.21, 0.79)	0.25	0.05 (− 0.25, 0.35)	0.75	− 0.04 (− 0.47, 0.40)		0.87
Net use	− 0.04 (− 0.10, 0.01)	0.09	− 0.06 (− 0.13, 0.0)	0.06	− 0.00 (− 0.07, 0.07)	1.00	− 0.02 (− 0.10, 0.06)		0.67

Data was calculated using linear regression analysis. A positive coefficient implies an increase in antibody levels. A negative coefficient implies a decrease in antibody levels. *P* values of < 0.05 were considered statistically significant

^**#**^Primigravid as the control

^**+**^Chikwawa as the reference variable

*SP as the control variable

*CI* confidence interval, *Coeff* Coefficient, *IgG* immunoglobulin G, *MUAC* mid-upper arm circumference

By contrast, in women who were uninfected at enrolment, levels of IgM at enrolment were higher in secundigravid and primigravid than in multigravid women, with no significant difference between primigravid and secundigravid women (*p* = 0.58) (Fig. 2B). In secundigravid women, infection at enrolment was associated with a small, non-significant reduction in ln IgM antibodies at enrolment (Coeff = − 0.14, 95% CI − 0.45 to 0.17, *p* = 0.37) (Table 3).

**Table 3:** Factors that may affect levels of in IgM antibody to DBL1X-ID2a VAR2CSA at enrolment and delivery

	ln IgM antibody at enrolment				ln IgM antibody at delivery			
	Infected at enrolment Coeff (95% CI)	*p-*value	Uninfected at enrolment Coeff (95% CI)	*p*- value	Infected at enrolment Coeff (95% CI)	*p-* value	Uninfected at enrolment Coeff (95% CI)	*p-* value
^#^Secundigravid	− 0.14 (− 0.45, 0.17)	0.37	0.23 (− 0.29, 0.75)	0.38	0.16 (− 0.18, 0.50)	0.36	0.05 (− 0.38, 0.48)	0.81
^#^Multigravid	− 0.16 (− 0.46, 0.13)	0.27	− 0.37 (−0.81, 0.06)	0.09	0.17 (− 0.15, 0.49)	0.30	− 0.17 (− 0.54, 0.20)	0.36
Maternal age (years)	− 0.13 (− 0.64, 0.38)	0.63	− **1.18 (**− **2.09, **− **0.26)**	**0.01**	0.34 (− 0.27, 0.95)	0.28	− 0.53 (− 1.67, 0.61)	0.36
Maternal height (cm)	− 0.14 (− 0.81, 0.54)	0.70	0.13 (− 0.75–1.00)	0.78	− 0.38 (− 1.19, 0.44)	0.37	0.17 (− 0.92, 1.26)	0.76
MUAC (cm)	− 0.13 (− 0.42, 0.16)	0.39	− **0.60 (**− **1.02, **− **0.18)**	**0.005**	− 0.18 (− 0.47, 0.12)	0.24	− **0.64 (**− **1.18, **− **0.11)**	**0.02**
^+^Madziabango	0.28 (− 0.10, 0.66)	0.15	0.40 (− 0.27, 1.06)	0.24	− 0.26 (− 0.66, 0.14)	0.21	− 0.04 (− 0.62, 0.54)	0.89
^+^Mangochi	− 0.20 (− 0.62, 0.22)	0.34	0.24 (− 0.38, 0.86)	0.44	− **0.71 (**− **1.19, **− **0.24)**	**0.003**	− 0.12 (− 0.69, 0.44)	0.66
^+^Mpemba	0.19 (−0.37, 0.75)	0.50	**0.63 (0.12, 1.14)**	**0.02**	− 0.20 (− 0.70, 0.31)	0.44	− 0.13 (− 0.27, 0.54)	0.52
^+^Zomba	− 0.07 (− 0.39, 0.24)	0.65	0.46 (− 0.11, 1.02)	0.11	− **0.50 (**− **0.83, **− **0.16)**	**0.004**	− 0.16 (− 0.63, 0.32)	0.52
DP	− 0.14 (− 0.45, 0.17)	0.37	− 0.14 (− 0.60, 0.31)	0.54	0.16 (− 0.18, 0.50)	0.36	− **0.49 (**− **0.86, **− **0.13)**	**0.009**
DP + AZ	− 0.16 (− 0.46, 0.13)	0.27	− 0.17 (− 0.65, 0.31)	0.51	0.17 (− 0.15, 0.49)	0.30	− 0.25 (−0.63, 0.14)	0.20
Net use	0.01 (− 0.04, 0.06)	0.61	− **0.07 (**− **0.14, **− **0.00)**	**0.05**	0.05 (− 0.005, 0.11)	0.07	− 0.03 (−0.12, 0.05)	0.45

Data was calculated using linear regression analysis. A positive coefficient implies an increase in antibody levels. A negative coefficient implies a decrease in antibody levels. *P* values of < 0.05 were considered statistically significant

^**#**^Primigravid as the control

^**+**^Chikwawa as the reference variable

*SP as the control variable

*CI* confidence interval, *Coeff* Coefficient*, IgM* immunoglobulin M, *MUAC* mid-upper arm circumference

### Other factors that affect the IgG and IgM antibody levels at enrolment and delivery

In women infected at enrolment, every cm increase in height was associated with a 0.98 decrease in IgG antibody at delivery (− 0.98 (− 1.87, − 0.09), *p* = 0.03) (Table 2).

At enrolment in uninfected women, increasing age was associated with lower IgM antibodies (coefficient − 1.18 (− 2.09, − 0.26), and each centimetre increase in MUAC was associated with a decrease in IgM antibody by approximately 60% (0.60 (− 1.02, − 0.18); *p* = 0.005) (Table 3). Bed net use in the same group of women was associated with 7% decrease in IgM levels at enrolment (− 0.07 (− 0.14, − 0.00); *p* = 0.05) (Table 3).

### Antibody levels at enrolment and association with protection against adverse pregnancy outcomes

Whether IgG or IgM antibody responses at enrolment were predictive of adverse pregnancy outcomes was investigated using univariate and multivariate logistic regression. There were no statistically significant associations observed between ln IgG antibody levels at enrolment and adverse birth outcomes, including LBW, SGA, PTD, or maternal anaemia, regardless of the pregnant woman's infection status at enrolment (Table 4A).

**Table 4:** Association between in IgG and IgM antibodies against DBL1X-ID2a VAR2CSA at enrolment and adverse pregnancy outcome

A. IgG		n	OR (95% CI)	*p*-value	n	aOR (95% CI)	*p*-value
Infected at enrolment	LBW	265	1.18 (0.81–1.67)	0.37	264	1.16 (0.81–1.67)	0.41
	Maternal anaemia	260	1.09 (0.85–1.40)	0.51	259	1.08 (0.84–1.40)	0.55
	PTD	273	1.25 (0.84–1.87)	0.27	272	1.24 (0.81–1.88)	0.32
	SGA	262	0.96 (0.74–1.25)	0.78	261	0.96 (0.74–1.25)	0.75
Uninfected at enrolment	LBW	129	0.71 (0.42–1.21)	0.21	127	0.76 (0.44–1.30)	0.31
	Maternal anaemia	136	0.82 (0.58–1.17)	0.28	134	0.80 (0.55–1.16)	0.23
	PTD	136	1.15 (0.61–1.97)	0.61	136	1.18 (0.68–2.05)	0.55
	SGA	125	0.99 (0.70–1.41)	0.97	123	1.08 (0.74–1.57)	0.71
B. IgM							
Infected at enrolment	LBW	265	1.16 (0.84–1.61)	0.36	258	1.14 (0.82–1.61)	0.42
	Maternal anaemia	260	**0.78 (0.61–1.00)**	**0.05**	259	**0.77 (0.60–0.99)**	**0.04**
	PTD	273	1.08 (0.76–1.54)	0.65	272	1.06 (0.73–1.54)	0.74
	SGA	262	1.00 (0.77–1.28)	0.98	261	1.00 (0.77–1.30)	0.99
Uninfected at enrolment	LBW	129	0.92 (0.54–1.55)	0.75	126	0.79 (0.46–1.35)	0.40
	Maternal anaemia	136	1.30 (0.90–1.87)	0.16	133	1.20 (0.81–1.77)	0.37
	PTD	136	1.03 (0.59–1.82)	0.91	133	0.92 (0.48–1.79)	0.81
	SGA	125	0.86 (0.59–1.25)	0.43	122	0.89 (0.60–1.31)	0.55

Unadjusted odds ratio (OR) and 95% confidence intervals (CI) determined by univariate logistic regression analysis. Missing values were removed before analysis. aOR: Adjusted odds ratio determined by multivariate logistic regression, adjusted for maternal age, height, MUAC at enrolment and gravidity. *LBW* low birth weight (< 2500 g), *PTD* preterm delivery (< 37 weeks of gestation), *SGA* small for gestational age (< 10th percentile of the infant’s gestational age), *IgG and IgM* Immunoglobulin G and M

No significant associations were identified between ln IgM antibody levels at enrolment and most of the adverse birth outcomes. However, among women who were infected at enrolment, for each unit increase in ln IgM, a 23% decrease in the odds of maternal anaemia at delivery was found, with an adjusted odds ratio (aOR) of 0.77 (95% CI 0.60–0.99), *p* = 0.04 (Table 4B).

A higher ln IgG/IgM ratio was significantly associated with increased odds of maternal anaemia among women infected at enrolment (aOR 1.24, 95% CI 1.01–1.52; *p* = 0.04). This association was not observed for other adverse pregnancy outcomes in uninfected women or when antibody ratios measured at delivery were analysed (Supplementary Table 1).

### Antibody levels at delivery and association with protection against adverse pregnancy outcomes

Whether antibody response at delivery was associated with clinical outcomes at delivery was investigated. Among women uninfected at enrolment, there was a significant association between ln IgG antibody at delivery and reduced risk of LBW, aOR 0.43 (95% CI 0.19–0.97; *p* = 0.04) (Table 5A) and a trend towards reduced risk of preterm delivery associated with ln IgM antibody levels at delivery, aOR 0.41 (95% CI 0.17–1.02; *p* = 0.06) (Table 5B). There were no associations between IgG and IgM antibody levels at delivery and SGA and maternal anaemia.

**Table 5:** Association between in IgG and IgM antibodies against DBL1X-ID2a VAR2CSA at delivery and adverse pregnancy outcomes

A. IgG		n	OR (95% CI)	*p*-value	n	aOR (95% CI)	*p*-value
Infected at enrolment	LBW	239	1.30 (0.85–1.97)	0.23	239	1.43 (0.89–2.28)	0.14
	Maternal anaemia	239	1.09 (0.81–1.47)	0.56	239	1.07 (0.78–1.46)	0.69
	PTD	243	1.40 (0.89–2.20)	0.15	243	1.52 (0.91–2.54)	0.11
	SGA	237	1.02 (0.74–1.41)	0.89	237	1.02 (0.73–1.43)	0.90
Uninfected at enrolment	LBW	129	**0.47 (0.23–0.98)**	**0.04**	127	**0.43 (0.19–0.97)**	**0.04**
	Maternal anaemia	133	1.04 (0.69–1.55)	0.86	131	1.11 (0.71–1.73)	0.65
	PTD	131	0.99 (0.52–1.86)	0.97	129	0.64 (0.28–1.48)	0.64
	SGA	126	0.97 (0.65–1.45)	0.88	124	1.09 (0.71–1.68)	0.70
B. IgM							
Infected at enrolment	LBW	238	1.28 (0.90–1.83)	0.18	238	1.36 (0.92–2.00)	0.12
	Maternal anaemia	238	0.88 (0.67–1.16)	0.36	238	0.85 (0.64–1.12)	0.25
	PTD	242	1.17 (0.79–1.72)	0.43	242	1.21 (0.79–1.84)	0.38
	SGA	236	1.03 (0.77–1.38)	0.82	236	1.03 (0.77–1.39)	0.83
Uninfected at enrolment	LBW	129	1.00 (0.53–1.92)	0.99	126	0.93 (0.48–1.80)	0.82
	Maternal anaemia	133	1.04 (0.67–1.62)	0.87	130	0.96 (0.60–1.52)	0.86
	PTD	131	**0.41 (0.17–0.95)**	**0.04**	128	0.41 (0.17–1.02)	0.06
	SGA	126	0.87 (0.56–1.35)	0.54	123	0.91 (0.57–1.46)	0.70

Unadjusted odds ratio (OR) and 95% confidence intervals (CI) determined by univariate logistic regression analysis. Antibody expressed as ln. Missing values were removed before analysis. aOR: Adjusted odds ratio determined by multivariate logistic regression, adjusted for maternal age, height, MUAC at enrolment and gravidity. *LBW* low birth weight (< 2500 g), *PTD* preterm delivery (< 37 weeks of gestation), *SGA* small for gestational age (< 10th percentile of the infant’s gestational age), *IgG and IgM* Immunoglobulin G and M

## Discussion

This study investigated whether pregnant women living in a malaria-endemic region who were exposed to the parasite during pregnancy have naturally acquired IgG and IgM antibodies against DBL1X-ID2a VAR2CSA at 16–28 weeks of gestation and delivery, and whether these antibodies are associated with protection from adverse pregnancy outcomes and maternal anaemia. To evaluate this hypothesis, plasma samples were collected from a cohort of 466 pregnant women living in malaria-endemic Malawi who were infected at some point during their pregnancy and associations were explored between the naturally acquired IgG and IgM antibody response to a recombinant DBL1X-ID2a VAR2CSA and adverse pregnancy outcomes.

This study shows that women infected at enrolment had higher levels of VAR2CSA-specific antibodies compared to those uninfected at this time. This is consistent with previous studies reported from Benin [11], Papua New Guinea (PNG) [14], and Mozambique [26], which showed that infection is associated with an increased level of IgG antibodies to recombinant DBL1X-ID2a VAR2CSA. IgG and IgM antibody levels decreased between 16 and 28 weeks and delivery in women infected at enrolment, suggesting that IgG and IgM antibody responses to DBL1X-ID2a VAR2CSA may be short-lived among infected women. This result is consistent with a study conducted on pregnant Malian women in which recombinant DBL1X-ID2a VAR2CSA-specific IgG levels were found to decrease after infection [27]. In malaria-infected pregnant Cameroonian women who were not receiving IPTp, IgG antibody responses to the ID1-ID2a antigen of VAR2CSA, as measured by Luminex assay, showed no significant change throughout pregnancy [28]. This observation is inconsistent with the present study, which demonstrated a change in antibody levels during pregnancy; the lack of variation in antibody levels in the Cameroonian cohort may reflect differences in malaria transmission intensity, exposure to IPTp, host immune factors, or antigenic variation in local parasite populations, highlighting the complexity of immune responses to malaria during pregnancy.

Levels of IgG antibody were gravidity-dependent, increasing from primigravid to multigravid women. In contrast, among women who were uninfected at enrolment, the levels of IgM antibody were negatively associated with gravidity, with higher levels observed in primigravid and secundigravid women compared to multigravid women (Fig. 2). IgM is the initial antibody produced in reaction to an infection, suggesting a primary immune system response [29]. Elevated IgM levels in primigravid and secundigravid women suggest that these individuals are undergoing initial or early exposures to *P. falciparum* during pregnancy. By contrast, multigravid women are more likely to have been previously exposed to malaria, which may explain why they produce more IgG than IgM antibodies to DBL1X-ID2a VAR2CSA.

Gravidity and maternal age were positively associated with IgG levels. In contrast, maternal age, height, MUAC and net use were negatively associated with IgM antibodies. Other studies have also reported that gravidity [11, 27] or age [30] affect antibody levels. This suggests that the ability to develop and maintain protective antibodies against malaria may be influenced by both the level of exposure to malaria parasites and the number of pregnancies a woman has had. In some research on malaria, low maternal nutrition, as measured by MUAC, has been associated with dampened antibody responses to malaria parasites [31, 32]. The present study instead suggests that well-nourished women have lower levels of antibodies; better-nourished women may be more affluent and better protected from malaria. Furthermore, a study conducted in Malawi during the second trimester (14–26 weeks of gestation) showed that the use of bed nets was associated with lower antibodies [33], highlighting the dynamics of the relationship between efforts to prevent malaria and the immune responses to it.

The majority of the research demonstrating a link between anti-VAR2CSA antibodies and better birth outcomes has focused on specific subsets of infected women [34, 35] or particular gravidity groups [14]. A comprehensive meta-analysis investigating associations between anti-VAR2CSA antibody responses and birth outcomes yielded inconsistent findings regarding their protective effects against adverse outcomes [16]. These variations indicate the difficulty in distinguishing antibodies that are markers of exposure from those that are markers of protection, highlighting the importance of further investigations to clarify the underlying mechanisms.

DBL1X-ID2a VAR2CSA-specific IgG measured at enrolment was not associated with a risk of preterm delivery, SGA, LBW or maternal anaemia; however, there was weak evidence that IgG at delivery was associated with a reduced risk of LBW in the subset of women who were uninfected at enrolment. Similarly, a study conducted in Benin identified an association between IgG3 against ID1-ID2a and a reduced risk of LBW, although no association was observed with other pregnancy outcomes [15]. In Benin, high levels of DBL1-2 specific IgG antibodies in pregnant women were not associated with PTD or maternal anaemia at delivery but were associated with reduced risk of LBW [11], while a study in Mali reported that VAR2CSA specific IgG antibody levels were not linked to lower risk of SGA and LBW [27]. In contrast, a longitudinal study conducted in Madang, PNG found that VAR2CSA-specific IgG antibody levels measured at enrolment in infected pregnant women were associated with improved birth weight [14]. In the present study, while IgG antibody levels at enrolment were not associated with protection against LBW, PTD, SGA or maternal anaemia, the association between elevated IgG to a DBL1X-ID2a VAR2CSA domain at delivery with a reduced risk of LBW points to the possibility that immune responses are involved in the determination of pregnancy outcomes and that immune modulation might be of particular importance in the later stages of pregnancy, though this association may be due to chance. Studies of the relationship between antibodies to VAR2CSA and levels of maternal haemoglobin or protection from anaemia have yielded varying results. Studies conducted in various African countries, including Malawi [36] and southern Mozambique [37] have not reported associations between antibodies to *VAR2CSA* and maternal haemoglobin or protection from maternal anaemia. In Cameroon, antibodies to recombinant VAR2CSA were associated with increased risk of maternal anaemia in pregnant women [38]. Other studies conducted in Malawi [39–42] have shown that various antibody measures to pregnancy-associated malaria variant surface antigens (VSA_PAM_) were associated with reduced risks of maternal anaemia.

While IgM antibodies targeting merozoites have been reported to lower the risk of clinical malaria in children in Papua New Guinea [43], limited research has explored their association with protective outcomes in malaria during pregnancy. In Mozambican pregnant women, IgM antibodies to non VAR2CSA *P. falciparum* antigen DBLα and lysate of 3D7, R29 and E8B parasite lines were reported to serve as markers of exposure rather than as a correlate of protection against maternal anaemia at delivery [26]. IgM antibodies to the VAR2CSA ID1-ID2a region were detected in pregnant Cameroonian women but were not associated with protection against placental malaria [28]. In the current study, IgM antibody levels to the VAR2CSA domain DBL1X-ID2a were not associated with reduced risk of LBW or SGA. At enrolment in infected women, IgM antibody levels were associated with reduced risk of maternal anaemia. At delivery, IgM antibody levels in women who were uninfected at enrolment were associated with a borderline lower risk of PTD. Though these findings may be due to chance and need to be confirmed in other cohorts, they suggest that interventions that elevate IgM antibody levels, possibly through vaccination, could be a viable strategy for reducing adverse outcomes associated with malaria in pregnancy. IgM antibodies may contribute to protection by promoting phagocytosis [44, 45].

The most notable strength of this work lies in the longitudinal nature of this study, which allowed examination of the relationship between the presence of antibodies mid-pregnancy and adverse pregnancy outcomes measured at delivery. The second strength lies in the number of participants enrolled (n = 466), from most of whom plasma samples collected at enrolment and delivery were available, permitting investigation of the change in antibody levels over pregnancy. One limitation of the study is the use of recombinant DBL1X-ID2a VAR2CSA antigen instead of full length VARCSA or CSA binding IE. Antibodies that recognize recombinant VAR2CSA domains might not be directed against the same epitopes that are targeted by antibodies associated with protection [46]. This may not allow for the assessment of conformational antibody epitopes only available in the quaternary structure of the full protein.

Future studies on this cohort could focus on elucidating IgA subclass responses and their possible functional roles in protection against placental malaria as the level of IgA2 to VAR2CSA DBL2 mid-pregnancy was associated with protection from placental malaria in a cohort of pregnant women from PNG [47]. IgA2 responses have also been correlated with effector functions such as antibody-dependent cellular phagocytosis (ADCP) and neutrophil activation, suggesting a potential role in clearing IEs or modulating inflammatory responses [48]. Similarly, measuring IgG subclasses in our cohorts would be valuable, given that cytophilic IgG3 has been consistently associated with higher birth weights, while elevated IgG4 levels have been linked to a reduced risk of placental infections [15]. Investigating IgM and IgG responses to full-length or native VAR2CSA could clarify associations with adverse pregnancy outcomes. Functional assays, such as phagocytosis of opsonized IEs and adhesion-blocking tests, may provide useful measures of functional immunity. Additionally, measuring afucosylated IgG using GlYcoLISA [49] or a fucose-sensitive enzyme-linked immunosorbent assay [50] could help, as PfEMP1-specific IgG is highly afucosylated and linked to NK cell activation and protection from LBW [51].

## Conclusion

Malaria infection increases IgG and IgM antibody levels against DBL1X-ID2a VAR2CSA in pregnancy. IgG antibody responses to the DBL1X-ID2a VAR2CSA recombinant protein may primarily be markers of malaria infection, while IgM antibody responses could also be markers of protection against maternal anaemia at delivery, warranting further investigation in other cohorts. Further research is required to identify antibody responses that serve as reliable markers of correlates of protection against adverse pregnancy outcomes.

Finally, IgM has been largely overlooked in vaccine research compared to IgG, and this study highlights the need to emphasize its measurement in malaria vaccine development and in studies of malaria antibody.

## Supplementary Information

Supplementary material 1.

Supplementary material 2.

## Data Availability

The data is available upon request from the corresponding author.

## Abbreviations

aOR: Adjusted odds ratio
AZ: Azithromycin
Cat no: Catalogue number
CI: Confidence interval
CSA: Chondroitin sulfate A
DBL: Duffy-Binding-Like
DP: Dihydroartemisinin-piperaquine
Hb: Haemoglobin concentration
IE: Infected erythrocytes
IgG: Immunoglobulin G
IgM: Immunoglobulin M
LBW: Low birth weight
LSTM: Liverpool School of Tropical Medicine
MiP: Malaria in pregnancy
MUAC: Mid-upper arm circumference
OD: Optical density
OR: Odds ratio
*P.** falciparum*: *Plasmodium falciparum*
PM: Placental malaria
PPS-IgG: Pooled positive sera IgG
PPS-IgM: Pooled positive sera IgM
PTD: Preterm delivery
qPCR: Quantitative polymerase chain reaction
RT: Room temperature
SD: Standard deviation
SGA: Small for gestational age
SP: Sulfadoxine-pyrimethamine
TMB: 3, 3’,5, 5’-Tetramethylbenzidine
