# Supplementary figures and images for: VAR2CSA-specific IgG and IgM antibodies are markers of exposure and protection against adverse malaria pregnancy outcomes
Source: Malar J. 2025 Dec 31;25:72. doi: 10.1186/s12936-025-05773-0 (PMC12866454; doi:10.1186/s12936-025-05773-0)

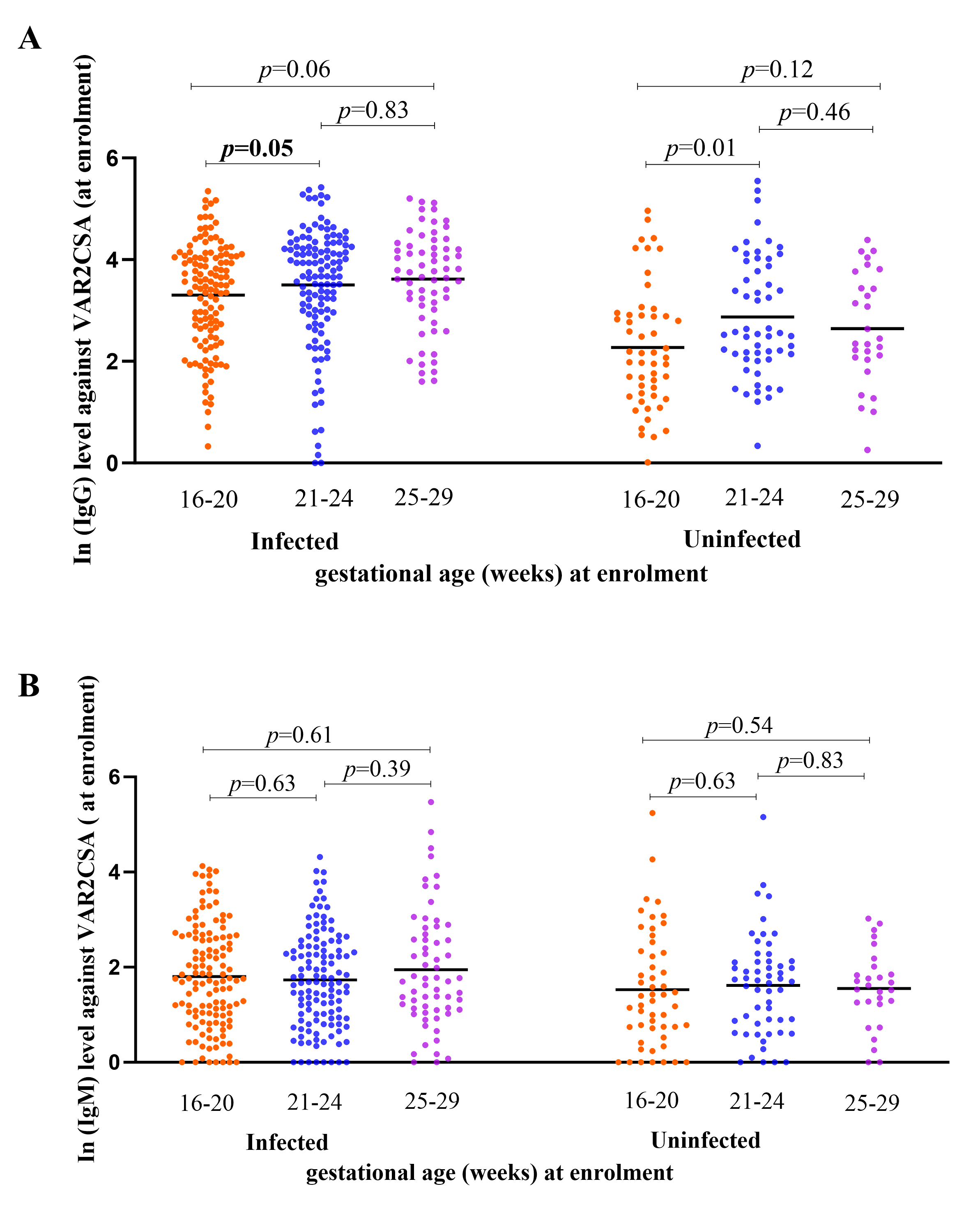

Supplement: Supplementary file 1 — Supplementary material 1. [file 12936_2025_5773_MOESM1_ESM.tif]
