# Supplementary material for: VAR2CSA-specific IgG and IgM antibodies are markers of exposure and protection against adverse malaria pregnancy outcomes
Source: Malar J. 2025 Dec 31;25:72. doi: 10.1186/s12936-025-05773-0 (PMC12866454; doi:10.1186/s12936-025-05773-0)
Supplement: Supplementary file 2 — Supplementary material 2. [file 12936_2025_5773_MOESM2_ESM.docx]

Supplementary table 1: Association of the ln IgG/IgM ratio to DBL1X–ID2a VAR2CSA at enrolment and delivery with adverse pregnancy outcomes.

|  | | | | | | | |
| --- | --- | --- | --- | --- | --- | --- | --- |
| **Enrolment** |  | **n** | **OR (95% CI)** | ***p*-value** | **n** | **aOR (95% CI)** | ***p*-value** |
| **Infected at enrolment** | LBW | 265 | 1.00 (0.77, 1.29) | 0.99 | 264 | 1.00 (0.77, 1.31) | 0.97 |
|  | Maternal anaemia | 260 | **1.23 (1.01, 1.50)** | **0.03** | 259 | **1.24 (1.01, 1.52)** | **0.04** |
|  | PTD | 273 | 1.07 (0.81, 1.43) | 0.61 | 272 | 1.08 (0.80, 1.46) | 0.60 |
|  | SGA | 262 | 0.98 (0.80, 1.20) | 0.86 | 261 | 0.98 (0.80, 1.20) | 0.83 |
| **Uninfected at enrolment** | LBW | 129 | 0.84 (0.55, 1.28) | 0.42 | 126 | 0.96 (0.62, 1.50) | 0.87 |
|  | Maternal anaemia | 136 | **0.72 (0.53, 0.98)** | **0.04** | 133 | 0.74 (0.53, 1.03) | 0.07 |
|  | PTD | 136 | 1.11 (0.69, 1.79) | 0.68 | 133 | 1.22 (0.72, 2.06) | 0.45 |
|  | SGA | 125 | 1.10 (0.81, 1.50) | 0.53 | 122 | 1.17 (0.82, 1.65) | 0.39 |
| **Delivery** |  |  |  |  |  |  |  |
| **Infected at enrolment** | LBW | 236 | 0.97 (0.71, 1.31) | 0.83 | 236 | 0.97 (0.70, 1.33) | 0.84 |
|  | Maternal anaemia | 236 | 1.14 (0.91, 1.42) | 0.24 | 236 | 1.14 (0.92, 1.44) | 0.23 |
|  | PTD | 240 | 1.07 (0.77, 1.48) | 0.68 | 240 | 1.08 (0.76, 1.53) | 0.66 |
|  | SGA | 234 | 0.99 (0.78, 1.26) | 0.94 | 234 | 0.98 (0.77, 1.25) | 0.90 |
| **Uninfected at enrolment** | LBW | 129 | 0.68 (0.42, 1.09) | 0.11 | 126 | 0.69 (0.41, 1.16) | 0.16 |
|  | Maternal anaemia | 133 | 1.00 (0.74, 1.37) | 0.98 | 130 | 1.08 (0.77, 1.52) | 0.67 |
|  | PTD | 133 | 1.42 (0.87, 2.33) | 0.16 | 130 | 1.39 (0.71, 2.71) | 0.34 |
|  | SGA | 126 | 1.05 (0.78, 1.42) | 0.76 | 123 | 1.09 (0.79, 1.51) | 0.60 |
|  |  |  |  |  |  |  |  |

Unadjusted odds ratio (OR) and 95% confidence intervals (CI) determined by univariate logistic regression analysis. Antibody expressed as ln. Missing values were removed before analysis. aOR: Adjusted odds ratio determined by multivariate logistic regression, adjusted for maternal age, height, MUAC at enrolment and gravidity. LBW: low birth weight (<2500g), PTD: preterm delivery (< 37 weeks of gestation), SGA: small for gestational age (<10^th^ percentile of the infant’s gestational age), IgG and IgM: Immunoglobulin G and M.
